# Portal biliopathy presenting with biliary stricture and bleeding: Role of digital cholangioscopy

**DOI:** 10.1055/a-2854-6963

**Published:** 2026-04-29

**Authors:** Emad S. Aljahdli

**Affiliations:** 1 Gastroenterology Division, Faculty of Medicine 37848 King Abdulaziz UniversityJeddahSaudi Arabia; 2 Gastrointestinal Oncology Unit 48132 King Abdulaziz University HospitalJeddahSaudi Arabia

Benign biliary strictures most commonly result from sclerosing cholangitis, choledocholithiasis, iatrogenic bile duct injury, infection, chronic pancreatitis, or congenital anomalies. Vascular-related causes, including portal hypertensive biliopathy, are less common. This rare condition is characterized by cholangiographic abnormalities due to portal cavernoma and periportal collaterals. Ischemic changes and extrinsic compression may lead to strictures, choledocholithiasis, and cholangitis. While cross-sectional imaging (ultrasound, computed tomography, magnetic resonance imaging [MRI], and endoscopic ultrasound [EUS]) may suggest the diagnosis, cholangioscopy offers superior accuracy for evaluating indeterminate biliary strictures. The introduction of digital single-operator cholangioscopy (DSOC) provides high-definition imaging and a wide endoscopic field of view, allowing real-time diagnostic assessment 1 .

**Fig. 1:**
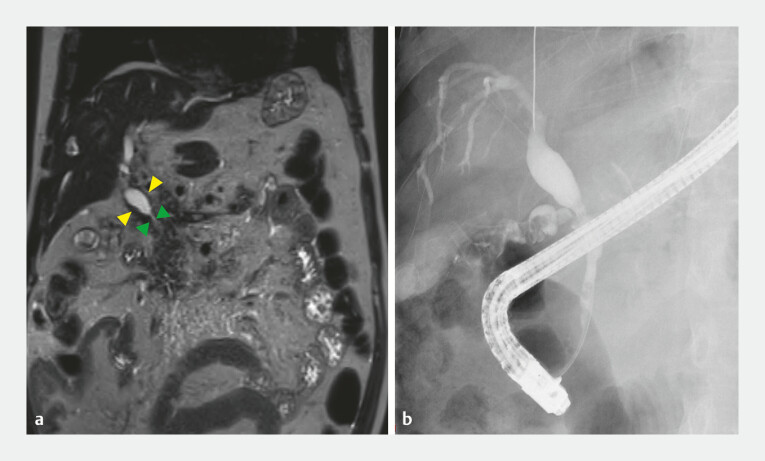
**a** Magnetic resonance cholangiopancreatographic and **b** ERCP cholangiogram images revealing the dilated proximal common bile duct (CBD) with a distal stricture. ERCP, endoscopic retrograde cholangiopancreatography.

A 55-year-old man with schistosomiasis-related chronic liver disease and portal hypertension presented with jaundice and intermittent abdominal pain. Laboratory tests showed elevated serum bilirubin levels. MRI demonstrated proximal common bile duct (CBD) dilatation with distal narrowing, without evidence of pancreatic masses ( Fig. 1 **a** ) Upper endoscopy revealed small, non-bleeding gastroesophageal and duodenal varices. EUS confirmed distal CBD narrowing with two intraductal stones, and a normal-appearing ampullary region. Endoscopic retrograde cholangiopancreatography successfully extracted the stones; however, the distal biliary stricture persisted ( Fig. 1 **b** ).

**Fig. 2:**
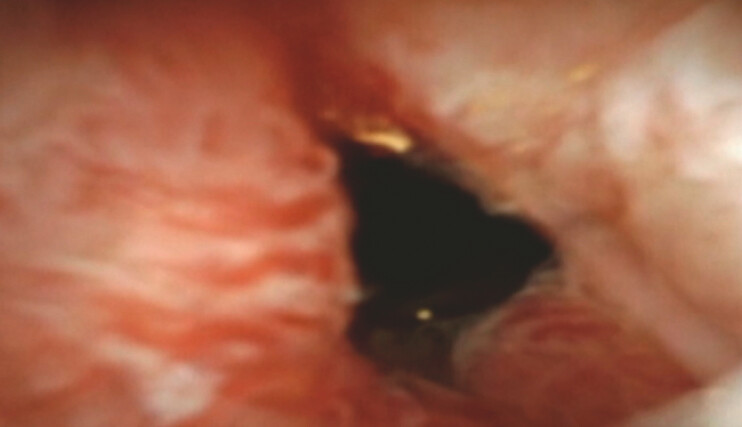
A digital single-operator cholangioscopic image revealing the mucosal lining of the distal CBD involving high-risk varices with red signs.

**Fig. 3:**
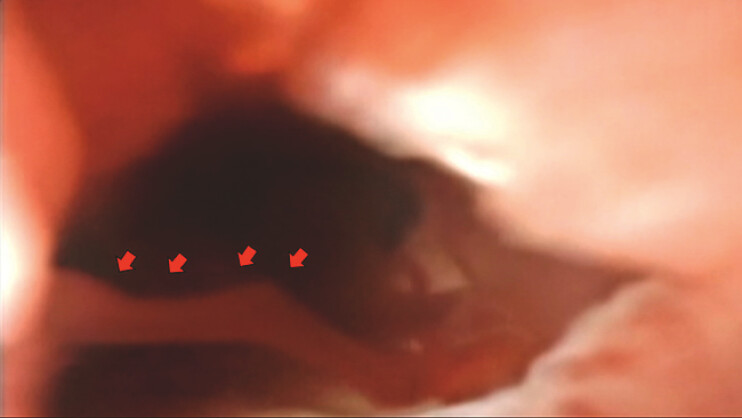
A digital single-operator cholangioscopic image revealing high-risk varices with active fresh bleeding (red arrows).

**Fig. 4:**
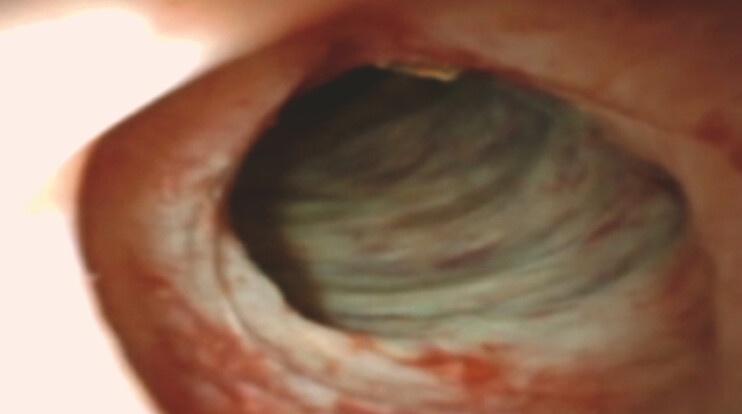
A digital single-operator cholangioscopic image revealing the flattening of the intraductal varices under the pressure effect of water irrigation.

DSOC revealed high-definition views of the distal CBD narrowing ( Video 1 ). Importantly, spontaneous active bleeding from mucosal varices with red signs was identified ( Fig. 2 and Fig. 3 ). The varices flattened after irrigation with water, confirming their vascular nature ( Fig. 4 ). A plastic biliary stent was initially placed, securing hemostasis and adequate drainage. This was later exchanged for a fully covered self-expandable metal stent. The patient was referred to interventional radiology for transjugular intrahepatic portosystemic shunt placement as a bridge to potential liver transplantation.

Digital single-operator cholangioscopy revealed a distal CBD stricture with spontaneous active bleeding from mucosal varices with red signs. Varices flattened upon water irrigation, confirming their vascular nature.Video 1

Portal biliopathy should be considered in patients with portal hypertension and indeterminate biliary strictures. DSOC provides invaluable, high-definition imaging that can differentiate vascular lesions from malignant or inflammatory strictures, guiding safe management and avoiding potentially hazardous interventions, such as brush cytology or forceps biopsy, in the setting of suspected vascular lesions.

Endoscopy_UCTN_Code_CCL_1AZ_2AZ
